# Draft genome of *Pseudomonas* sp. XK-1, a marine bacterium capable of degrading lignin and Mn(II) oxidation

**DOI:** 10.1128/mra.00411-24

**Published:** 2024-06-12

**Authors:** Xukang Wang, Haixia Pan, Hao Zhou, Zhongqing Feng, Ang Li, Xiaoyan Guan

**Affiliations:** 1 Key Laboratory of Industrial Ecology and Environmental Engineering (Ministry of Education), School of Chemical Engineering, Ocean and Life Sciences, Panjin Campus, Dalian University of Technology, Panjin, China; 2 State Key Laboratory of Urban Water Resource and Environment, School of Environment, Harbin Institute of Technology, Harbin, China; 3 Liaoning Ocean and Fisheries Science Research Institute, Dalian, China; Indiana University, Bloomington, Bloomington, Indiana, USA

**Keywords:** *Pseudomonas*, genome, carbon cycling, manganese oxidation

## Abstract

We report the draft genome sequence of marine bacteria, *Pseudomonas* sp. XK-1. Strain XK-1 could facilitate Mn(II) oxidation with lignin as the sole carbon source. The genome length of XK-1 is 4,751,776 bp, with a G + C content of 62.61%. Genome analyses reveal the carbon and manganese cycling driven by bacteria.

## ANNOUNCEMENT

Most species within the genus *Pseudomonas* are aerobic heterotrophs, and some possess the ability to oxidize Mn(II) (1–4). However, complex carbon source utilization capacity of them was poorly reported. In this study, we isolated an Mn(II)-oxidizing bacterium from nearshore marine sediments that was initially identified as *Pseudomonas* by 16S rRNA gene sequencing and designated as XK-1. Strain XK-1 could grow with lignin as the sole carbon source, which suggests its ability to degrade lignin.

Sediment samples at the surface layer were grabbed randomly with a Peterson mud picker and stored at 4°C in 500 mL sterile PE bottles. The strain XK-1 was isolated by incubating in an enrichment medium at 30°C and streaking on agar plates repeatedly. The medium contains NaCl (1 g/L), MgCl_2_ (0.4 g/L), CaCl_2_ (1 g/L), KCl (5 g/L), Na_2_SO_4_ (0.142 g/L), K_2_HPO_4_ (0.026 g/L), NaNO_3_ (0.085 g/L), supplemented with MnCl_2_ (1 mM), alkali lignin (2 g/L), and agar (20 g/L if necessary). After several runs of enrichment and purification, the pure brown colonies were obtained and turned blue after dropping with Leucoberbelin blue, a specific dye that reacted with Mn(III) and Mn(IV). Additionally, the degradation of lignin by XK-1 was confirmed by the decrease of total organic carbon, which decreased from 450 mg/L to 230 mg/L within 48 h (5, 6).

Prior to DNA extraction, the strain was incubated in enrichment medium for 7 days (7). Extractions were performed using an Sodium chloride-Tris-EDTA (STE) method and quantified with Qubit fluorometer. Then, sequencing was performed by Novogene Co. Ltd. (China) with a DNA library prepared using a Novogene NGS DNA library Prep Set. Subsequently, the genome sequencing was performed using Illumina NovaSeq platform (PE150). Default parameters were used for all software unless otherwise specified. The raw reads underwent trimming with Trimmomatic v0.40 and quality checking using FastQC v0.12.1 (8, 9). The assembly was performed using SOAP *de novo* (Version 2.04), and fragments below 500 bp were filtered out (10). The assembly was first annotated with NCBI’s PGAP 6.6 (11). The functional genes were annotated using the Kyoto Encyclopedia of Genes and Genomes database (12). The genome sequence was uploaded to the Type (Strain) Genome Server (TYGS) server (https://tygs.dsmz.de/) for digital DNA-DNA hybridization (dDDH) analysis and identification (13).

After trimming, totally 8,073,334 high-quality reads were obtained. The assembled genome of strain XK-1 contains 12 contigs with 254× coverage, the maximum contig was 1,280,430 bp, the N50 was 562,023 bp, and the N90 was 182,938 bp. The resulting genome of strain XK-1 was 4,751,776 bp with a G + C content of 62.61%. The annotation results show that a total of 4,291 coding genes were predicted, and 56 tRNAs, 3 rRNAs (1 × 5S, 1 × 16S, and 1 × 23S), and 4 ncRNAs were identified. The result of dDDH shows that the best matching strain of XK-1 is *Pseudomonas anguilliseptica* DSM 12111 with the dDDH (d_4_) of 29.2%. Therefore, strain XK-2 was classified as the genus of *Pseudomonas* (14).

## Data Availability

The draft genome of Pseudomonas sp. XK-1 has been submitted to NCBI database under BioProject number PRJNA1092820 and BioSample number of SAMN40636090 has been deposited in the GenBank (No. JBBPES000000000). The illumina sequence reads have been deposited in the Sequence Read Archive (SRA) database under the accession number SRR28714109.
